# Ultrasonic Evaluation of the Adhesion Between Regenerative Coatings and Steel Substrates

**DOI:** 10.3390/ma19112313

**Published:** 2026-05-29

**Authors:** Jakub Kowalczyk, Marian Jósko, Waldemar Matysiak

**Affiliations:** 1 Faculty of Civil and Transport Engineering, Institute of Machines and Motor Vehicles, Poznan University of Technology, 61-131 Poznan, Poland; marian.josko@put.poznan.pl; 2 Faculty of Mechanical Engineering, Institute of Materials Technology, Poznan University of Technology, 61-131 Poznan, Poland; waldemar.matysiak@put.poznan.pl

**Keywords:** coatings, regeneration, circular economy, ultrasonic testing

## Abstract

The rise in resource consumption and environmental pressures are driving the development of a circular economy, in which the remanufacturing of machine and vehicle parts plays a significant role. Despite numerous studies on regeneration technologies (including additive manufacturing, cladding, and spraying), the literature lacks analyses concerning the non-destructive evaluation of the coating–substrate bond quality—when tested from the substrate side. The aim of this study was to evaluate the applicability of the ultrasonic method for the detection of defects (delaminations, detachments) in the adhesive joint between the regenerative coating and the base material. The study demonstrated that the ultrasonic method enables non-destructive, portable, and single-sided inspection of the integrity of the coating–substrate interface, making it a useful tool in industrial practice. Fifteen samples (including one reference sample) of varying thicknesses (from 1.9 µm to 1753.9 µm) were tested using ultrasonic probes with frequencies ranging from 1 MHz to 20 MHz. It was found that the adhesion of coatings approximately 74 µm thick can be estimated using an ultrasonic wave with a frequency of 1.66 MHz, whereas for coatings approximately 250 µm thick, adhesion can be estimated using higher frequencies, i.e., from 2.75 to 20 MHz.

## 1. Introduction

The growing consumption of natural resources, increasing amounts of waste, and climate change are leading to the circular economy model becoming increasingly common [1,2]. The most widely accepted definition of the circular economy is that it is a self-renewing and regenerative economy [3,4]. To regenerate and rebuild the economy, many factors must be considered, one of which is the remanufacturing of machine and vehicle parts. The use of remanufactured parts not only reduces repair costs but also has an important environmental aspect; they also pose a logistical challenge [5]. The circular economy is leading to an increase in the number of companies engaged in the regeneration of parts [2,6]. During the inspection of machine parts, they are classified into one of three groups: fit for further use, suitable for repair (for example, by straightening or grinding), and unserviceable. Parts that are unsuitable for further repair may be remanufactured or scrapped, depending on the availability of remanufacturing methods and the parts themselves.

In practice, various regeneration methods are used. One of the newest methods is regeneration using additive manufacturing. An interesting case of the regeneration of a damaged slide valve removed from a hydraulic distributor is presented; it was repaired using additive manufacturing (AM) technology based on laser powder bed fusion (PBF-LB/M) [7]. The authors demonstrated that during a tensile test, the hydraulic manifold slide remanufactured using the SLM method achieved a stress level of 450 MPa. In the same test, the original slide achieved a value of 1000 MPa. The interface between the base material and the remanufactured material achieved a hardness of 630 HV. The M300 maraging steel, which served as the remanufactured material, achieved a hardness of 390 HV, while the base material reached 260 HV. Although the work was very interesting, the authors did not present an analysis of the possibilities for non-destructive quality control of the remanufactured area. The topic of using additive technologies in the remanufacturing process was also addressed in other studies [8,9,10]. These studies also did not address the issue of non-destructive evaluation of the work performed, which is important from a practical standpoint.

Another study [11] provides an interesting discussion of brake caliper regeneration. The authors devoted the main part of their work to the issue of part flow. This study also completely omitted the issue of non-destructive testing of the work performed.

Typical technical aspects of regeneration were examined, for example, in [12], where the regeneration of an engine crankshaft was studied; an iron-based coating on 45 steel was obtained using laser cladding. This study examined the properties of the cladding layers. It was observed that the layer and the substrate exhibit good metallurgical bonding; however, unfortunately, the issue of non-destructive testing was not addressed here either. In another study [13], the feasibility of repairing crankshaft journals using the cold spraying method was investigated. It was demonstrated that the size and shape of the repair are limited by both the efficiency of the cold spraying process and the design of the crankshaft, but the issue of non-destructive evaluation of the bond between the coating and the base material was not addressed.

One of the challenges in the regeneration of machine and vehicle parts using regenerative coatings is the evaluation of the coating–substrate adhesive bond. Among the available methods for evaluating adhesive bonds, it was decided to use the ultrasonic method. This method was chosen because it is fast, fully portable, allows for the testing of components made of various materials and different coatings, and requires access to the material under testing from only one side.

The ultrasonic method was used to test adhesive bonds for various materials, including aluminum. In [14], the ultrasonic method in pulse-echo mode was applied to inspect individual aluminum adhesive bonds containing interphase defects in the bonded area. The authors demonstrated that examining the first interface reflection has the lowest probability of accurately determining the defect size, but the presence of a defect is identified. For sizing, the second and third interface reflections show better performance for inclusions and delaminations, respectively.

Various technical configurations are used in the ultrasonic testing of adhesive joints. These are based on measuring the difference in the amplitude of signals reflected from the joint [15,16]. The ultrasonic method was also used to examine adhesive bonds [17]. It was demonstrated that ultrasonic testing of adhesive (glue) joints at an oblique angle showed that adhesive degradation can be measured based on significant changes in the reflection amplitude, as well as a shift in the minimum of the reflection spectrum.

The analysis conducted, along with numerous other studies, confirms the need for non-destructive evaluation of repair coatings. One of the main challenges is the testing method. Most studies are conducted from the coating side and make the significant simplification that the wave penetrates the entire thickness of the repair coating [18]. The authors demonstrated that the use of ultrasonic transducers with a water delay line allows for the classification of adhered and delaminated coating layers with an accuracy of over 99%. Based on this study, the thickness and adhesion state of the coating layer can be easily determined by combining a waveform spectrogram with a CNN network. These studies focused on specific coatings, namely those used in marine environments.

It was also demonstrated that metal coatings applied by thermal spraying protect steel structures from corrosion and extend their service life; however, over time, they themselves may degrade, crack, and lose their protective properties A continuous monitoring system using FBG sensors was developed, which allows for real-time detection of the rate and progression of corrosion as well as the formation of cracks in the coating [19].

Studies on the quality of the coating–substrate bond have also been conducted in other works. The authors demonstrated that it is possible to perform both quantitative and qualitative assessments of the bond; however, unfortunately, the study was limited to a single type of coating, namely the body filler coating used in the restoration of automotive vehicle bodies [20]. The authors demonstrated that the amplitude of the Rayleigh ultrasonic wave pulse is a useful diagnostic parameter for determining the thickness of the body filler coating on a steel automotive body substrate. As the thickness of the applied putty increases, the amplitude of the surface wave pulse passing through the adhesive joint decreases (from 99% of the ultrasonic flaw detector’s screen height in the absence of a coating to 15.3% for a 13 mm thick coating).

There is a clear need to use non-destructive testing methods in the evaluation of adhesive bonds, as defects such as delamination, separation, discontinuities, or so-called “kissing bonds” may be internal and not visible during visual inspection, yet still significantly affect the load-bearing capacity and durability of the bond. For this reason, the literature emphasizes the need to use non-destructive testing methods, such as ultrasonic, radiographic, or thermographic testing, particularly for joints critical to the structure [21].

Certainly, the need to use non-destructive testing methods for assessing the quality of coating–substrate interfaces is increasingly emphasized, since defects such as delamination, debonding, or local interfacial discontinuities may not be visible during visual inspection yet may still significantly affect the durability and functionality of the coating system [22,23,24].

The main objective of this study was to evaluate the potential of ultrasonic defetoscopy in assessing the adhesion of regenerative coatings to the substrate, specifically their presence, continuity, adhesion, and structural integrity. The use of the ultrasonic method stems from its anticipated high efficiency, safety, and full mobility, as well as the ability to conduct tests with access from only one side of the component. The tests were conducted from the side opposite to the coated surface. This approach enables a non-destructive assessment of the coating’s condition and its adhesion to the substrate, while simultaneously providing real-time feedback on the quality of the bond, without the need to disassemble selected components and machine parts.

## 2. Research Methodology

### 2.1. Research Plan

**Figure 1:**
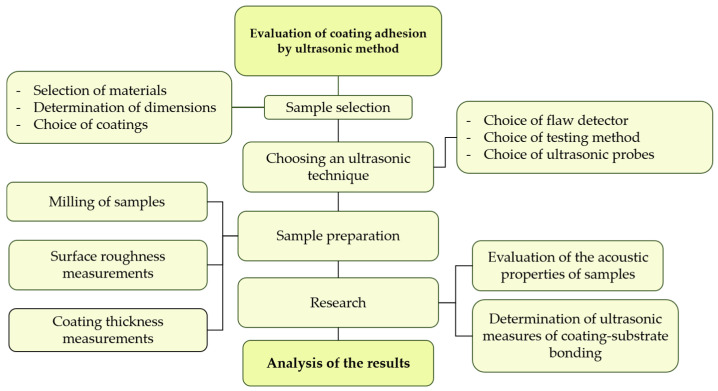
The full research plan.

To achieve the set objective, research was planned and conducted. All work was carried out in accordance with the plan shown in Figure 1. First, the test material was selected, followed by the testing equipment. Next, the shape and dimensions of the test specimens were determined, as well as the types of coatings. After preparing the samples, they were checked to ensure they all had similar attenuation, and ultrasonic tests were conducted. The full work plan is shown in Figure 1.

### 2.2. Characteristics of the Materials Studied

First, the material for preparing the samples was selected. Since steel consistently accounts for a significant proportion of the materials used in the manufacturing of machine and vehicle parts [25], it was decided to use 1.0503 carbon steel for the samples. This is an unalloyed medium carbon steel. The chemical composition of the steel used is presented in Table 1.

Carbon steel 1.0503 has a tensile strength (Rm) ranging from 560 to 850 MPa, a yield strength ranging from 275 to 490 MPa, a hardness after annealing of 229 HB or less, an elongation of 14 to 17%, and a reduction in area of 35 to 45%.

**Figure 2:**
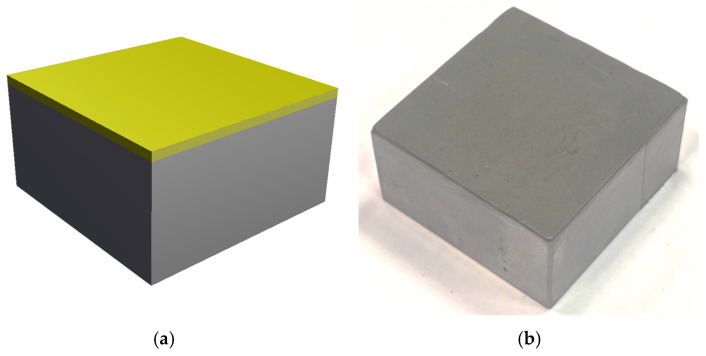
Samples used in the study; (**a**) model, (**b**) view of an actual sample.

To minimize measurement errors, it was decided that rectangular specimens would be used for testing, with the coating applied to a larger surface area. Under industrial conditions, ultrasonic probes with frequencies ranging from 2 to 10 MHz and a transducer diameter of approximately 6 mm are most commonly used for testing steel components. The highest testing accuracy is achieved for tests conducted outside the near field. Considering the available ultrasonic probes, a sample thickness of 25 mm was selected. To increase the accuracy of the tests, it was assumed that the prepared samples should allow measurements to be taken at at least sixteen independent points. Samples measuring 50 × 50 mm were selected for the tests. A model and photograph of an actual sample are shown in Figure 2.

**Figure 3:**
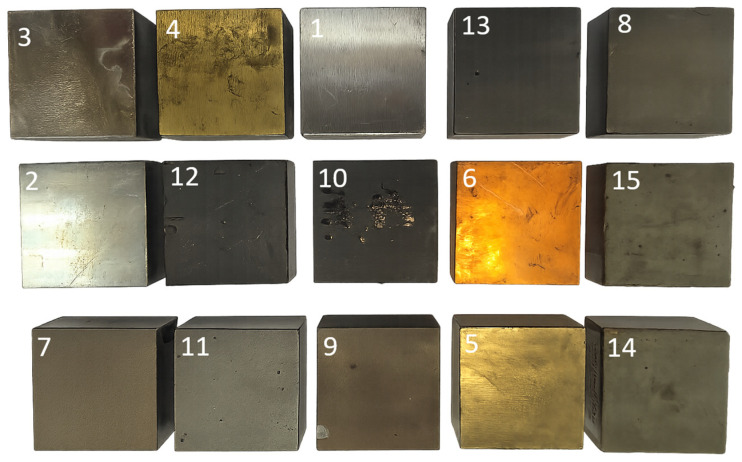
Set of samples used in the study (quantification from Table 2).

The study utilized 15 samples, one of which was a control sample that had not been coated. The samples were numbered 1 through 15, as shown in Table 2. A view of all the samples used is shown in Figure 3.

The coatings were prepared in accordance with the manufacturers’ technical specifications, and the thickness distributions of the applied coatings were examined for all samples.

**Figure 4:**
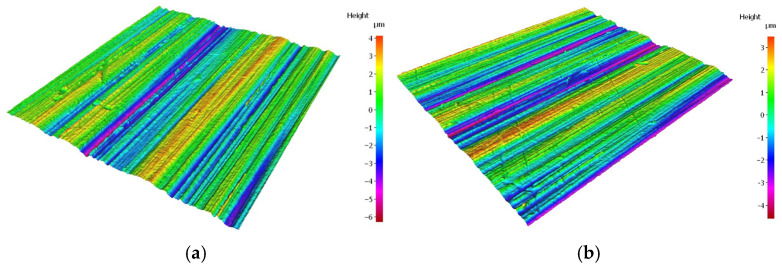
Surface roughness of the selected samples: (**a**) reference specimen, (**b**) specimen no. 1 surface.

Surface roughness was checked prior to coating application. Roughness was measured on both 50 × 50 mm surfaces. For all specimens, each surface was prepared in the same manner. Machining was performed on a CNC DMG MORI M1 milling machine (DMG MORI, Pleszew, Polnad) using a multi-edge cutter head equipped with cemented carbide inserts. Five surfaces were milled in a single clamping operation, which ensured the perpendicularity and parallelism of the faces. After milling, each surface was ground using a surface grinding machine. The purpose of these measurements was to assess the effect of surface condition on the propagation of the ultrasonic wave. The basic roughness parameter Ra for all samples, for both surfaces, ranged from 0.7 to 1.2 µm. A summary of the Ra parameter values for the tested samples is presented in Table 3. Figure 4 shows example results of 3D roughness measurements.

**Figure 5:**
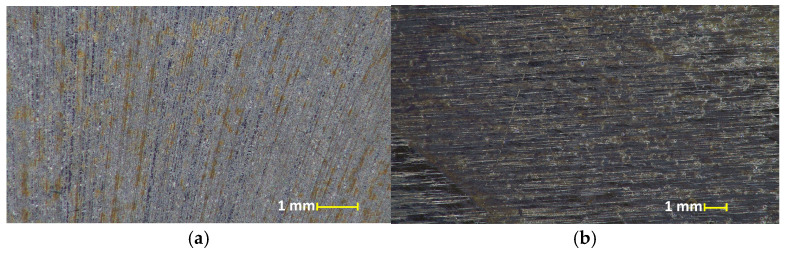

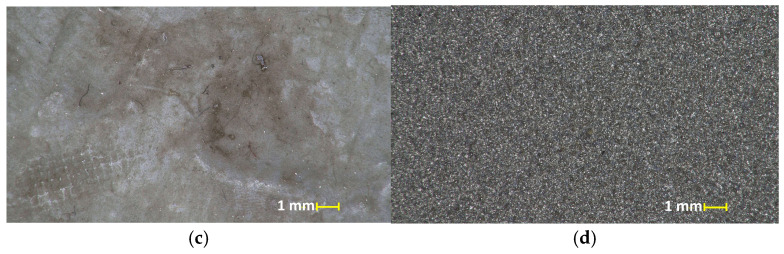
Examples of coating surface appearances of selected samples: (**a**) bare surface, (**b**) UNIREP 2 coating, (**c**) DevconFlexane General Purpose Putty coating—UT-cleaned; (**d**) P-51000 coating.

An example view of the surface before coating is shown in Figure 5a, while the applied surfaces of coatings are shown in Figure 5b,c.

### 2.3. Ultrasonic Examination Methods

In industrial settings, two basic methods of ultrasonic testing are used: the through-transmission method and the pulse-echo method. The through-transmission method requires the use of two ultrasonic transducers positioned opposite each other, whereas the pulse-echo method requires only a single ultrasonic transducer. Since it was assumed that the evaluation would focus on the adhesion of the coating to the base material on the side opposite to the side from which the testing is conducted, it was decided to use the ultrasonic normal incidence pulse-echo technique.

**Figure 6:**
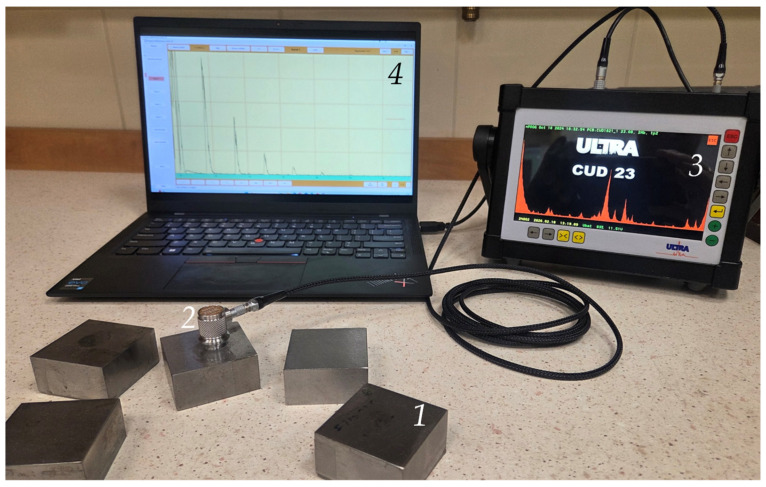
Test setup: 1—coated sample, 2—ultrasonic probe, 3—ultrasonic flaw detector, 4—computer (signal analysis).

A portable, digital ultrasonic flaw detector CUD (ZBM ULTRA sp. z o.o., Nadolice Male, Poland) was used for the tests. A diagram of the test setup is shown in Figure 6.

In ultrasonic testing, various ultrasonic parameters can be used to evaluate the adhesion of a coating to a substrate. Since the study focused on the substrate material, the through-transmission method and the use of surface waves were not considered in further analysis. For the ultrasonic pulse-echo technique, it is possible to use quality measures such as: the pulse height from the adhesion area, the pressure modulus of the reflection coefficient (total reflection, bottom echo, and three echoes), and the decibel drop in the height of the first two pulses obtained on the flaw detector screen.

**Figure 7:**
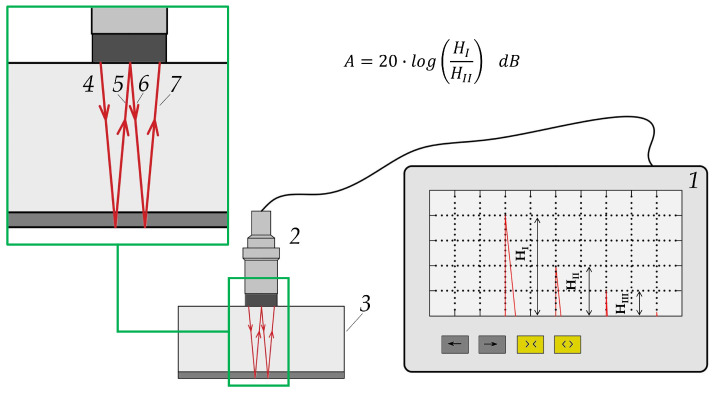
Test setup; 1—ultrasonic flaw detector, 2—ultrasonic probe, 3—sample, 4—transmitted wave pulse, 5—wave reflected from the coating, 6—wave reflected from the upper surface of the sample, 7—second pulse reflected from the bottom, A—decibel drop in pulse amplitude; H_I_, H_II_, H_III_—the first, second, and third pulses on the flaw detector screen, respectively.

Taking into account potential industrial applications, the decibel drop in the amplitude of the first two first pulses was selected for further work; the principle for determining this is shown in Figure 7 (the computer has been omitted to simplify the figure and improve readability). Measuring bond quality using this method is characterized by the lowest risk of errors resulting from the ultrasonic probe’s contact pressure on the tested material and the flaw detector’s settings, particularly the signal gain.

In the pre-studies, different ultrasonic probes were used, varying both the probe diameter and its frequency.

The list of ultrasonic probes and their parameters is presented in Table 4.

**Figure 8:**
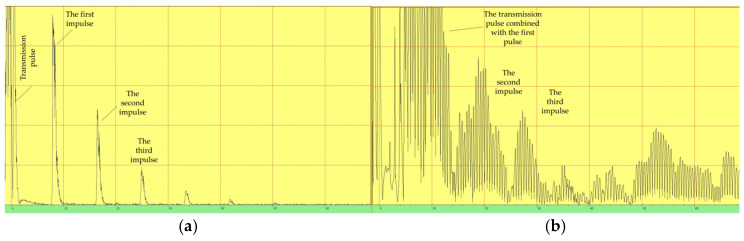
Signals displayed on the flaw detector screen: (**a**) suitable for ultrasonic testing with a 10 MHz probe; (**b**) unsuitable for ultrasonic testing with a 1 MHz probe.

First, we checked whether it was possible to obtain signals useful for the study on the flaw detector screen. For a probe with a frequency of 1 MHz and a probe diameter of 12 mm, no useful signal was obtained. Figure 8 shows a comparison of a useful (Figure 8a) and a useless (Figure 8b) signals on the flaw detector screen. Both signals come from the same sample but were obtained using different ultrasonic probes. Due to the inability to obtain signals useful for the study, probe 1L0 was excluded from further testing.

Thickness measurement coatings were performed using the Karl Deutsch KD2050 leptoscope (Karl Deutsch, Wuppertal, Germany) and PRODIG-TECH GL-PRO-6-FAZ (Prodig Tech, Bielsko-Biała, Poland).

## 3. Research Results

Before beginning the ultrasonic measurements, thickness measurements were taken for each individual coating. Thicknesses ranging from 1.9 µm to approximately 1754 µm were obtained. These differences stem from the fact that each coating was produced using a different manufacturing process. Table 5 presents the average thicknesses of the applied coatings along with the standard deviation of these thicknesses. The study also examined the thickness distributions within the samples. It was observed that the thicknesses for the individual manufacturing methods were similar to one another.

**Figure 9:**
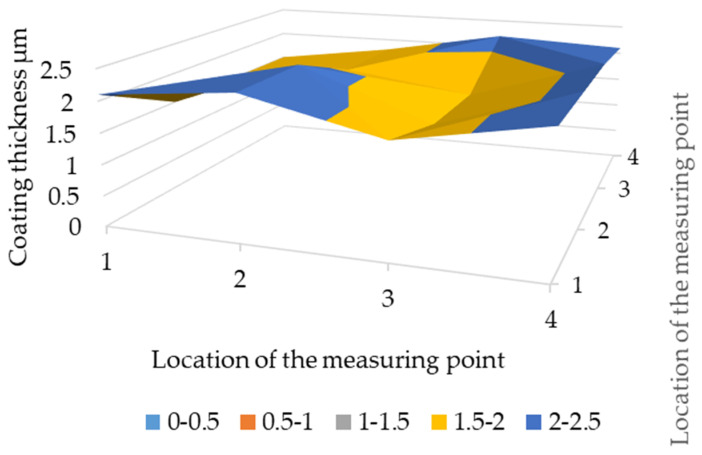
Thickness distribution of the zinc coating.

**Figure 10:**
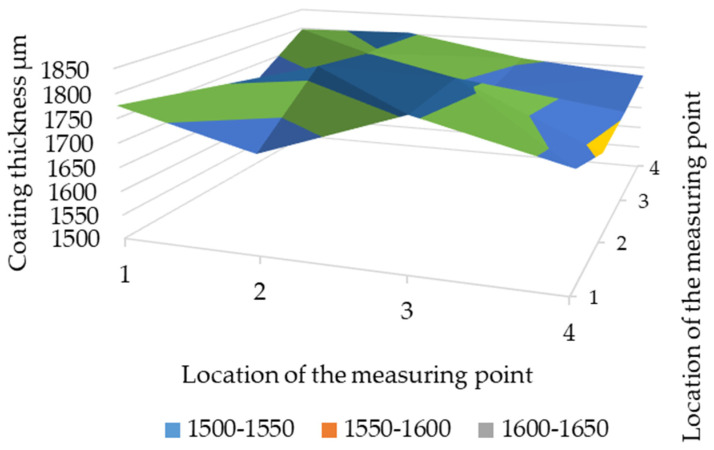
Thickness distribution of the DevconFlexane General Purpose Putty coating—sandblasted.

**Figure 11:**
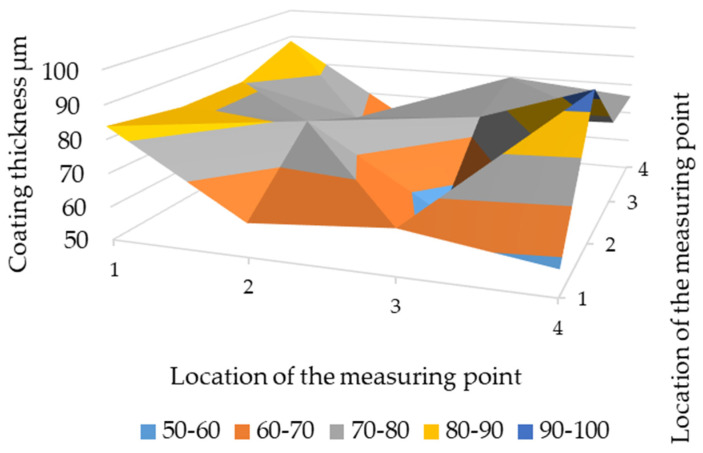
Thickness distribution of the P-51000 coating.

**Figure 12:**
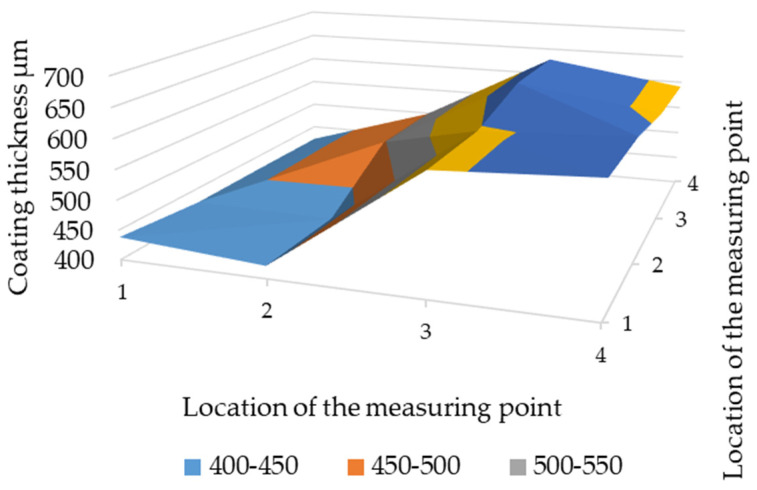
Thickness distribution of the UNIREP 3 coating.

Sixteen coating thickness measurements were taken on each sample. The measurement points were evenly distributed across the sample surface in a 4 × 4 grid pattern, corresponding to four rows and four columns of measurement points. This arrangement ensured uniform coverage of the analyzed surface and allowed for the acquisition of representative coating thickness values in various areas of the sample. Selected examples of coating thickness distributions are shown in Figure 9, Figure 10, Figure 11 and Figure 12.

The results of surface roughness measurements indicate that the surface condition of the tested samples has no significant effect on the propagation of ultrasonic waves, which allows us to consider its impact on the ultrasonic measurement results to be negligible [26,27].

Since the attenuation of the base material has a significant impact on the test results, it was verified whether there were differences in attenuation of ultrasound for all samples after the coatings were applied. Tests were also conducted before applying the coatings, but no change in the attenuation coefficient was observed. Initially, the measurement error for attenuation was determined by taking 30 measurements at a single point on the sample. The 95% confidence interval for the mean, calculated based on the relationship 1, was adopted as the error.

(1) $$\Delta_{\beta }=t_{0.95;n-1}\cdot \frac{s}{\sqrt{n}}$$

where the above signifies half-width of the 95% confidence interval, n—number of measurements, and s—standard deviation.

For the results obtained, a measurement error of 0.022 dB/mm was determined for the attenuation of ultrasonic wave 1.66 MHz frequency.

**Figure 13:**
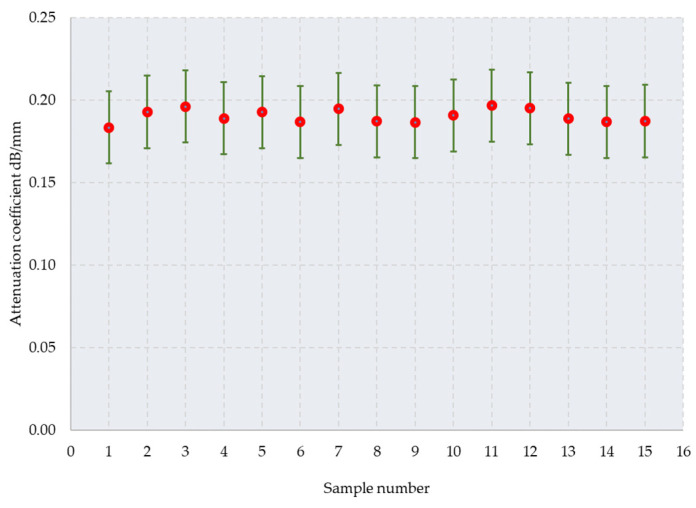
Damping coefficient of ultrasound for all tested samples.

The average attenuation values of ultrasound for all tested samples are shown in Figure 13. The results indicate that the attenuation of the native material is similar for all samples.

**Figure 14:**
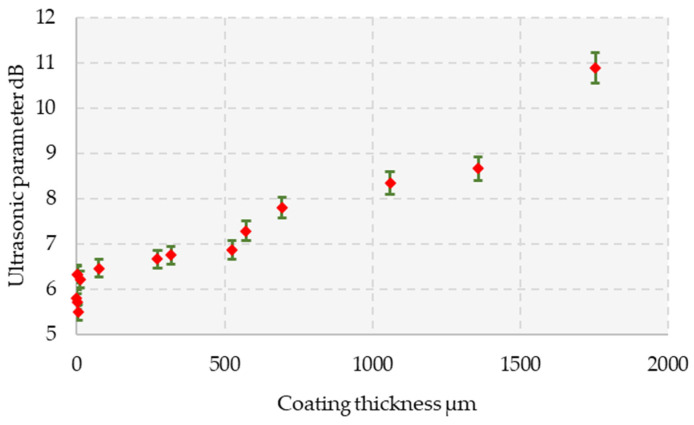
Changes in the decibel drop of the amplitude of two first pulses as a function of coating thickness for the DS12 HB 1–6 ultrasonic probe of 1.66 MHz frequency.

**Figure 15:**
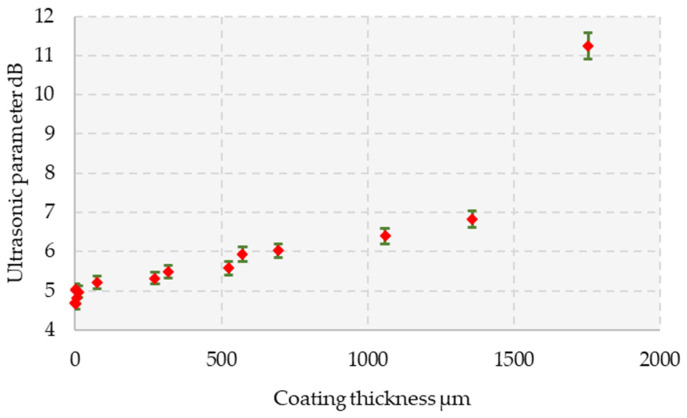
Changes in the decibel drop of the amplitude of two first pulses as a function of coating thickness for the MB2s ultrasonic probe of 2.775 MHz frequency.

**Figure 16:**
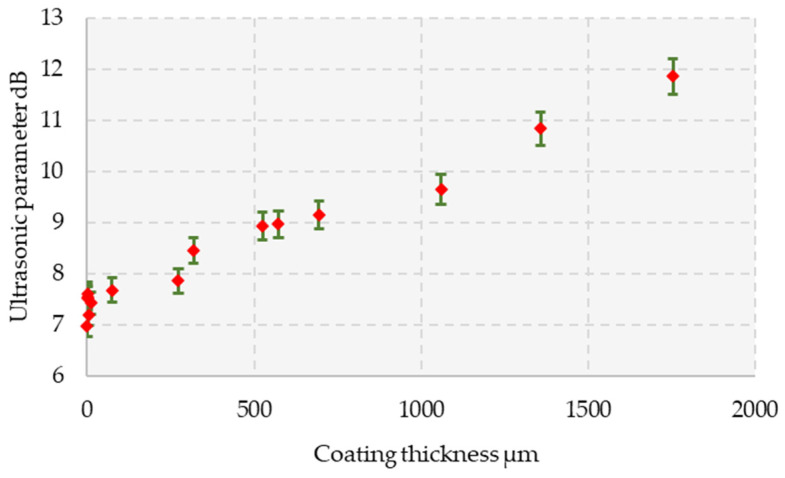
Changes in the decibel drop of the amplitude of two first pulses as a function of coating thickness for the MB4S ultrasonic probe of 4.45 MHz frequency.

**Figure 17:**
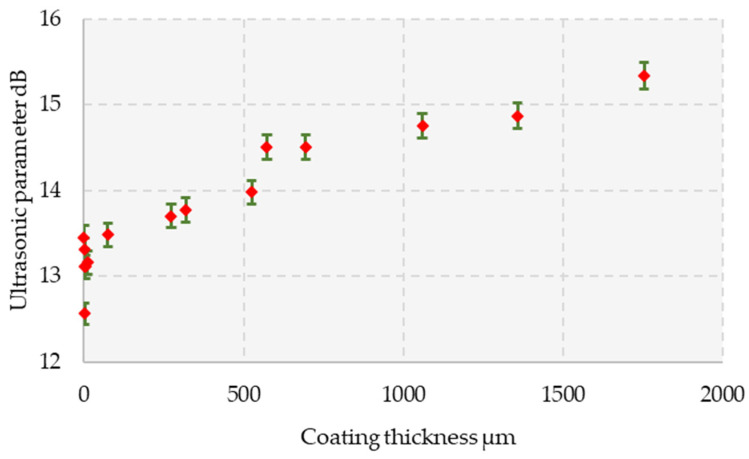
Changes in the decibel drop of the amplitude of two first pulses as a function of coating thickness for the P10 ultrasonic probe of 6.37 MHz frequency.

**Figure 18:**
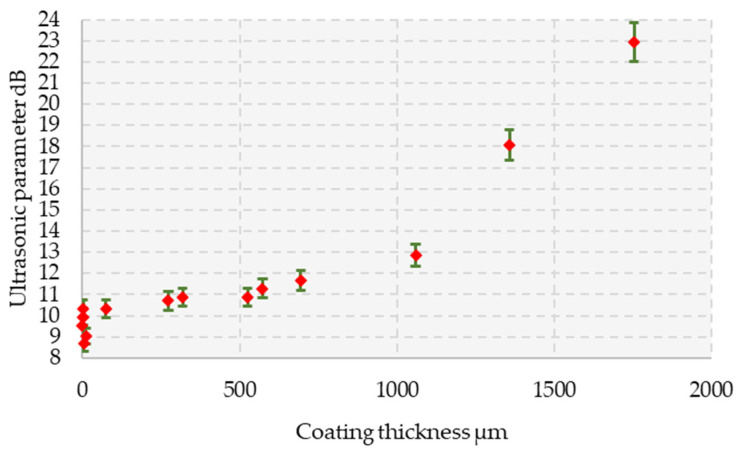
Changes in the decibel drop of the amplitude of two first pulses as a function of coating thickness for a 20 MHz ultrasonic probe of 20 MHz frequency.

In the next stage of the research, ultrasonic measurements were determined for all the ultrasonic probes used and for all the coatings analyzed. A summary of the results obtained is presented in Figure 14, Figure 15, Figure 16, Figure 17 and Figure 18.

The full set of results obtained in the studies are presented in Table 6.

Based on the results presented, it can be concluded that evaluating coatings with a thickness of less than 270 µm is difficult to perform using available ultrasonic probes. At the same time, it was noted that the differences between ultrasonic measurements for the reference sample and coated samples ranged from 20% to 230% for coating thicknesses above 270 µm. The largest differences were observed for the transducer with the highest frequency, which confirms the correlation with the feasibility of ultrasonic testing.

## 4. Discussion

Fifteen samples were subjected to ultrasonic testing, one of which was a reference sample without a coating. The thickness of the coatings was varied, in accordance with the manufacturers’ technical specifications. The coating thicknesses ranged from 1.9 µm to 1753.9 µm. Five samples had coatings thinner than 12 µm, two thinner than 100 µm, four thinner than 1 mm, and three samples thicker than 1 mm (Table 6).

It was observed that coating thickness affects the ultrasonic measurement materials of coatings. The study confirmed that the frequency of the longitudinal ultrasonic wave has a significant impact on the feasibility of ultrasonic examination of the adhesive bond.

The highest correlation coefficient between the tested coating and the ultrasonic wave frequency was found at 4.45 MHz (the correlation coefficient was 0.98). The lowest correlation coefficient was found for an ultrasonic transducer with a wave frequency of 2.75 MHz.

In ultrasonic testing, coating thickness has a significant effect on ultrasonic wave propagation. As coating thickness increases, the amount of energy absorbed due to wave attenuation also increases. This phenomenon is particularly important because ultrasonic wave attenuation in the coating material is significantly greater than in the steel substrate (Figure 14, Figure 15, Figure 16, Figure 17 and Figure 18). Consequently, thicker coatings attenuate the ultrasonic signal more strongly, which may affect the ability to interpret it unambiguously. At the same time, it was observed that the ultrasonic measures strongly depend on the ultrasonic transducers used. For the transducer with a frequency of 20 MHz, the range of the ultrasonic measure values was from 9.5 to 22.9 dB (Figure 18), whereas for the transducer with a frequency of 6.37 MHz, it ranged from 13.5 to 15.3 dB (Figure 17). This confirms that ultrasonic wave attenuation increases with increasing frequency [28,29].

At the same time, for thin coatings, the attenuation effect is relatively small, and changes in the ultrasonic signal may be difficult to unambiguously correlate with the actual coating thickness. Therefore, in such cases, ultrasonic testing may primarily allow for an approximate assessment of the presence of a coating on the material’s surface, while a reliable determination of its thickness may be limited or impossible.

For coatings with the smallest thickness, below 100 µm (for all considered coating materials), the correlation coefficients between coating thickness and ultrasonic measures are very low, amounting to approximately 0.5, regardless of the frequency and diameter of the ultrasonic transducer (Table 6).

The greatest differences in the decibel drop of the amplitude of the two first pulses were observed for the transducer with the highest frequency (20 MHz). A clear difference was observed for coating thicknesses above 270 µm, amounting to 20%; for the thickest coatings and a frequency of 20 MHz, the difference was 230% (Figure 18). It should be noted that the coatings used in the studies were produced in accordance with the established manufacturing technology, which ensured technological process repeatability within the given substrate preparation method.

The smallest scatter of the ultrasonic measure values was observed for the ultrasonic transducer with a frequency of 6.37 MHz (Figure 17), whereas the largest scatter was found for the transducers with frequencies of 2.78 MHz (Figure 15) and 20 MHz (Figure 18). This is important because it may provide practical guidance for planning ultrasonic tests. When testing elements characterized by high ultrasonic wave attenuation, such as cast iron components, it may be more appropriate to use a 6.37 MHz transducer. In contrast, when testing materials with lower attenuation, such as steel, or elements of small thickness, the use of a 20 MHz transducer may be more justified.

## 5. Conclusions

Based on the analysis of the research results, it can be concluded that the ultrasonic method can be used for the non-destructive evaluation of the bond between regenerative coatings and the substrate, with access only from the side of the base material. This confirms its potential applicability in industrial conditions, especially for fast, portable, and single-sided inspection of coated components.

The results showed that coating thickness has a significant influence on the ultrasonic signal parameters. For very thin coatings, especially below 100 µm, the relationship between coating thickness and ultrasonic measures was weak; therefore, in this range, the method may mainly allows only the approximate detection of the coating presence.

For thicker coatings, particularly above approximately 270 µm, clear changes in the ultrasonic parameter were observed, indicating the possibility of estimating the coating–substrate bond quality. The effectiveness of the method depends mainly on the coating thickness, coating material, and the frequency of the ultrasonic wave used.

## Figures and Tables

**Table 1:** Chemical composition of 1.0503 carbon steel (mass fractions in %).

C	Si	Mn	Cr	Ni	Mo	Cu	S	P
0.42	0.10	0.50	max	max	max	max	max	max
0.50	0.40	0.80	0.30	0.30	0.10	0.30	0.04	0.04

**Table 2:** List of coatings used in the tested samples.

Numbers	Coating
1	No coating—reference surface
2	Zinc coating
3	Nickel coating
4	Brass coating
5	Brass coating—with prior nickel plating
6	Copper coating
7	P-51000 coating
8	Devcon Special Epoxy coating
9	HA-7 coating
10	UNIREP 2 coating
11	Devcon Special F Epoxy coating
12	UNIREP 3 coating
13	Devcon Aluminum Liquid F-2 coating
14	Devcon Flexane General Purpose Putty coating—UT cleaned
15	Devcon Flexane General Purpose Putty coating—sandblasted

**Table 3:** Roughness parameter for all samples.

Roughness Parameter Ra µm															
No.	1	2	3	4	5	6	7	8	9	10	11	12	13	14	15
SA	1.2	1.2	0.7	0.8	1.0	1.0	1.1	0.9	1.0	1.1	0.7	0.9	1.0	1.1	0.9
ST	1.0	0.9	1.0	1.0	0.8	1.0	1.0	1.2	0.8	0.9	0.8	0.9	1.0	1.1	0.9

SA—the surface on which the coating was applied; ST—the surface from which the tests were carried out.

**Table 4:** Parameters of the ultrasonic probes used.

Parameter		1L0^O^12	DS12 HB 1–6	MB2S	MB4S	P10–10L	20 MHz
Probes designation		1L0	DS2	MB2	MB4	P10	20 MHz
Manufacturer		ULTRA	KD	GE	GE	KD	GE
Frequency	MHz	1	1.66	2.75	4.45	6.37	20
Diameter of the probe	mm	12	12	10	10	10	5
Effective diameter	mm	11.64	11.64	9.7	9.7	4.85	4.85
Wave speed of tested material	m/s	5948	5948	5948	5948	5948	5948
Wave length	mm	5.94	3.58	2.16	1.34	0.93	0.30
Near field	mm	4.2	8.6	10.3	17.3	25.0	19.7
The coefficient of decrease in decibels K	-	0.87	0.87	0.87	0.87	0.87	0.87
Distance from the probe	mm	25	25	25	25	25	25
beam width	mm	15.8	7.8	5.6	3.5	2.4	1.5

**Table 5:** Thicknesses of individual coatings.

Numbers	Coating	Average Thickness µm
1	No coating—reference surface	-
2	Zinc coating	1.9 ± 0.3
3	Nickel coating	2.1 ± 1.6
4	Brass coating	2.4 ± 1.6
5	Brass coating—with prior nickel plating	3.6 ± 0.4
6	Copper coating	11.5 ± 1.3
7	P-51000 coating	74 ± 11.1
8	Devcon Special Epoxy coating	273.1 ± 3.2
9	HA-7 coating	319.1 ± 29.2
10	UNIREP 2 coating	525 ± 85.4
11	Devcon Special F Epoxy coating	572.8 ± 15.8
12	UNIREP 3 coating	694 ± 4
13	Devcon Aluminum Liquid F-2 coating	1059.4 ± 6.1
14	Devcon Flexane General Purpose Putty coating—UT cleaned	1358.1 ± 29.4
15	Devcon Flexane General Purpose Putty coating—sandblasted	1753.9 ± 46.9

**Table 6:** Ultrasound measurement results.

Nr	Thickness	Decibel Drop in the Height of the Two First Pulses Obtained on the Flaw Detector Screen dB				
		1.66 MHz	2.75 MHz	4.45 MHz	6.37 MHz	20 MHz
1	0.0	5.81	4.68	6.98	13.45	9.54
2	1.9	5.72	4.67	7.51	12.57	9.95
3	2.1	6.33	5.01	7.55	13.31	9.95
4	2.4	6.33	5.03	7.60	13.11	10.33
5	3.6	5.49	4.81	7.19	13.11	8.67
6	11.5	6.22	4.98	7.42	13.16	9.05
7	74.0	6.45	5.22	7.68	13.48	10.33
8	273.1	6.66	5.33	7.86	13.70	10.70
9	319.1	6.75	5.49	8.46	13.77	10.88
10	525.0	6.87	5.58	8.92	13.98	10.88
11	572.8	7.29	5.93	8.96	14.51	11.29
12	694.0	7.80	6.02	9.15	14.51	11.67
13	1059.4	8.34	6.40	9.64	14.75	12.87
14	1358.1	8.66	6.83	10.83	14.87	18.06
15	1753.9	10.88	11.25	11.85	15.34	22.92

## Data Availability

The original contributions presented in this study are included in the article. Further inquiries can be directed to the corresponding author.
